# Persistent use of psychotropic drugs in nursing home residents in Norway

**DOI:** 10.1186/s12877-017-0440-5

**Published:** 2017-02-13

**Authors:** Anne-Sofie Helvik, Jūratė Šaltytė Benth, Bei Wu, Knut Engedal, Geir Selbæk

**Affiliations:** 1 Department of Public Health and General Practice, Faculty of Medicine, Norwegian University of Science and Technology (NTNU), Postboks 8905, NO-7491 Trondheim, Norway; 2 St Olavs University Hospital, Trondheim, Norway; 3 Norwegian National Advisory Unit on Ageing and Health, Vestfold Health Trust, Tønsberg, Norway; 4 Institute of Clinical Medicine, Campus Ahus, University of Oslo, Oslo, Norway; 5 HØKH, Research Centre, Akershus University Hospital, Lørenskog, Norway; 6 Centre for Old Age Psychiatric Research, Innlandet Hospital Trust, Ottestad, Norway; 7 Rory Meyers College of Nursing, New York University, New York, NC USA; 8 Institute of Health and Society, Faculty of Medicine, University of Oslo, Oslo, Norway

**Keywords:** ATC, Dementia, Older adults, Geriatrics, Long-term-use, Neuropsychiatric symptoms, Old age

## Abstract

**Background:**

The prevalence of psychotropic drug (PTD) use in NH residents is high, but few have explored prevalence and persistency in PTD in NH residents and factors associated with persistency. This at the same time as we know that risk of side events may be higher with long- term use in older adults. Thus, the aim of this study was to describe the prevalence and persistence in use of PTD and to explore factors associated with persistence in use of PTD at two consecutive time points in nursing home (NH) residents.

**Methods:**

We included 1163 NH residents in a 72-month longitudinal study with five assessments. Use of PTD, neuropsychiatric symptoms (NPS), severity of dementia and physical health were assessed each time.

**Results:**

The prevalence over time and persistent use of antipsychotic drugs, antidepressants, anxiolytics and sedatives at two consecutive time points were high in residents with and without dementia. There was an association between greater NPS at the first time point, and persistent use of these drugs, but changes in NPS between time points, did not explain such use. A longer NH stay increased the odds for persistent use of antipsychotics.

**Conclusion:**

Psychotropic drugs are frequently used as a long-term treatment among NH residents and are associated with severity of neuropsychiatric symptoms, but not with severity of dementia. Closer attention should be paid to follow-up of psychotropic drug treatment, and especially for long –term use of antipsychotics, since the duration of such treatment should be as short as possible.

## Background

The use of psychotropic drugs in European nursing homes (NH) is reported to be quite common. The prevalence of use of any psychotropic drug (PTD) varied between 42 and 80% in studies published from 2005 to 2013 [1–6] and varied between countries [7, 8]. Among NH residents with dementia or cognitive impairment, the prevalence of any PTD use is similar or even higher than among those with normal cognition (48–90%) [9–14] and also higher than in community living older adults with dementia [14]. In NHs in Scandinavia, the use of any PTD is also high (57–80%) [15–19], especially in studies that only include residents with dementia (68–85%) [12, 15, 17, 20, 21].

Over the years there has been an increase in use of PTD in NH [12, 18, 22–28] except for antipsychotic drugs, where a decrease has been observed in recent years [18, 27, 29, 30]. Thus, the prevalence of antipsychotic drug use in Scandinavian NH residents with dementia is among the lowest in Europe (pooled estimates 24%) [31]. Antidepressants are the most commonly used PTD in Norwegian NH residents [16, 18, 22].

Antipsychotic drugs are often used to treat neuropsychiatric symptoms, such as aggression, agitation or psychotic symptoms in NH residents with dementia, although non-pharmacological interventions should be the first choice of treatment for these symptoms [32, 33]. However, in demanding clinical situations, antipsychotic drugs may be unavoidable, but there is no evidence that long-term use of antipsychotic drugs in the management of neuropsychiatric symptoms is effective [33]. Older NH residents with dementia can be withdrawn from long-term antipsychotics without detrimental effects on their behavior, but caution is required in residents with severe neuropsychiatric symptoms [34]. Furthermore, the duration of antipsychotic drug treatment should be as short as possible, because of the high risk of side effects [32], such as more rapid progression of dementia [9] or cognitive decline [35], higher risk of cerebrovascular events [36] and increased risk of falling [37]. Use of antipsychotic drugs is also associated with increased mortality risk [38]. While the side effects of antipsychotics have received the most attention, studies show that there are serious short- and long-term side effects such as falls and fall-related fractures associated with use of antidepressants and with use of benzodiazepines [37]. The efficacy of antidepressants on depression in persons with dementia was not confirmed in a meta-analysis summarizing many randomized control trials [39]. A recent study of discontinuation of antidepressants in NH residents with dementia and neuropsychiatric symptoms in Norway found that most residents (85%) tolerated discontinuation [40]. However, when antidepressants are discontinued in residents with dementia they should be monitored carefully to identify those with worsening depressive symptoms [40]. Authors who studied retrospectively discontinuation of long-term use of benzodiazepines in older adults with and without dementia in care institutions found limited evidence of adverse outcomes due to discontinuation of benzodiazepines, but close attention should be paid to the possibility of emergent agitation and in patients with anxiety caution should be practiced [41]. Discontinuation of long-term use of Benzodiazepines in older adults without dementia has been found to increase handgrip strength, balance and cognitive function [42–44].

Of the approximately 75 studies on PTD in NH facilities published after 2004, only a small fraction have examined use of PTD in a longitudinal design [21, 45–54]. About half of the longitudinal studies have studied the prevalence of different types of PTD over time or persistent use at two time points [21, 45–48, 50, 52], but very few have studied factors associated with the persistent use of PTD, such as antipsychotic drugs [46, 52], antidepressants, anxiolytics or sedatives. However, as the risk of side events may be higher with long-term use [32, 33] and is decreased after discontinuation of PTD in older adults [34, 55] it is of vital importance to study the persistent use of PTD and its associated factors.

The aim of this study was to describe the prevalence and persistence of PTD use and to explore factors associated with the persistence in use of PTD at two consecutive time points in a Norwegian sample of NH residents.

## Method

### Design

This was a 72-month longitudinal study with five assessments. Baseline assessment (A1) took place between November 2004 and January 2005 [56]. The follow-up assessments took place after 12, 31 and 52 and 72 months (A2–A5).

### Setting and participants

In total, Norway has 40,000 NH places (beds) [57] for a population of about five million, with about 14% (700,000) of those aged 65 years or older [58]. The jurisdiction for public health care services lies with local municipalities, and local authorities offer social services (such as housing and home services), in-home nursing and institutional care (mainly in NHs), and provide both long- and short-term care and rehabilitation.

This study recruited participants from 26 NHs in 18 municipalities. The selection of small, medium, and large municipalities was made to obtain a wide variety of NH-settings in the sample. NH residents with a stay of at least 14 days were eligible for inclusion, no other inclusion or exclusion criteria were used [56]. In all, 1165 residents were eligible for inclusion and only two declined to participate.

### Measures

Psychotropic drugs were grouped according to the Anatomical Therapeutic Chemical (ATC) Classification System into the following groups: antipsychotics (N05A except lithium), antidepressants (N06A), anxiolytics (N05B), hypnotics/sedatives (N05C), and anti-dementia medication (N06D) (yes versus no) [59]. Combination drugs outside ATC NO5B and NO5Cwere not included. The information was collected from the medical record of each resident [56].

Dementia and severity of dementia was assessed using the Clinical Dementia Rating (CDR) scale, covering six domains (memory; orientation; judgment and problem solving; community affairs; home and hobbies; and personal care) with five response categories (0, 0.5, 1, 2, 3) [60, 61]. The total score was calculated using an algorithm that gives priority to memory [60]. Residents with a total score of one or higher were regarded as having dementia. The cut-off CDR ≥1 in defining dementia has been found adequate in previous Norwegian and international studies [62–64]. The categorical scores indicate the severity of dementia: a CDR score of 1 represents mild dementia, a CDR score of 2 represents moderate dementia, and a CDR score of 3 represents severe dementia. The sum-score of the six domains (sum of boxes), ranging from zero to 18, can also be used to measure the severity of dementia, as the categorical and continuous scores correlate highly ≥ 0.9 [65, 66]. The Spearman correlation between the categorical CDR score and the CDR sum of boxes score in the present study was 0.93. Due to a wider range of values, the CDR sum of boxes offers important advantages when analyzing the data [66].

We used the CDR score (CDR ≥1) as an indication of dementia, as it was not possible to perform a standardized dementia work-up for all residents because many were too frail or mentally impaired to take part in examinations such as CT or MRI. A large number of residents with a CDR score of 3 could not be tested with any dementia tests such as the Mini Mental Status Examination [67] or the Clock Drawing Test [68].

Physical health was assessed using the General Medical Health Rating (GMHR) scale [69]. This is a one-item global rating scale with four categories: good, fairly good, poor, and very poor. The rating was based on all available information on physical health and use of drugs. The scale has been used in large studies including older people with and without dementia [70] and has been translated and used in several studies [71, 72].

The Personal Activities of Daily Living (P-ADL) score was assessed with the Physical Self-Maintenance Scale (PSMS), including six items, with a total score ranging from 6 to 30 [73]. High scores indicate a lower level of functioning.

Neuropsychiatric symptoms (NPS) were assessed using the Neuropsychiatric Inventory Nursing Home version (NPI-NH) [74, 75]. The 10-item inventory covers the following symptoms: delusion, hallucination, euphoria, agitation/aggression, disinhibition, irritability/lability, depression/dysphoria, anxiety, apathy/indifference, and aberrant motor behavior (no/yes). For each symptom, severity (score 1–3) multiplied by frequency (score 1–4) provides a score from zero to 12. Based on a previous principal component analysis, psychosis (delusions, hallucination), agitation (agitation/aggression, disinhibition, irritability), and affective (depression, anxiety) sub-syndrome scores were formed by summing the score of the included items [76–79]. The apathy/indifference was analyzed as a single symptom.

Demographic information such as age, gender, marital status, and length of stay in the NH at the time of inclusion was collected from medical records. The type of unit was also recorded from among the following options: regular units (RU), special care unit for people with dementia (SCU), rehabilitation unit (REU), and other units (OU), mainly psychogeriatric wards. The length of stay in a NH before study inclusion was measured in days.

### Procedure

Nurses with extensive clinical experience collected the data. Prior to data collection, all assessors participated in a 2-day course on how to apply the standardized questionnaires. A 1-day training program was carried out prior to each follow-up assessment. The project leader (GS) was available for consultation throughout the data collection period. The nurses collected data from medical records and via a standardized interview with the residents’ primary caregivers, all of whom were registered nurses. All assessment scales used were standard translated Norwegian versions. A pilot study including 41 NH residents was carried out to test inter-rater reliability of CDR prior to the first data collection and the inter-rater reliability was very good. The kappa statistics for the global CDR score were 1 (between geriatric psychiatrist and registered nurse specialized in psychiatry) and 0.86 (between geriatric psychiatrist and registered nurse). More detailed information of the inter-rating reliability test has been published elsewhere [56].

Study information was given to the residents and their family members. The residents and their next of kin were informed that they could refuse to participate at any stage of the study. This procedure was recommended and approved by the Regional Ethics Committee in the south east of Norway, the Data Inspectorate and the Directorate for Health and Social Affairs in 2004 before data collection.

### Data analysis

Sample characteristics at baseline were described as means and standard deviations (SD) or frequencies and percentages. Residents with CDR ≥ 1 and CDR < 1 were compared by Independent samples t-test for continuous and χ^2^-test for categorical variables. Prevalence and persistence of PTD use among those with and without dementia were compared by Z-test for proportions. The persistence in outcomes, use of antipsychotics, antidepressants, anxiolytics and sedatives were assessed with a logistic regression model for hierarchical data (SAS GLIMMIX procedure). Random effects for type of unit nested in a NH were included into the model. The dependent variable was current use of a specific type of PTD drug, while the independent variable was either use of the same drug at the previous time point (lag 1), two time points previously (lag 2) or three time points previously (lag 3). All models were adjusted for a number of relevant covariates measured at the same time point as the independent variable. A similar model was estimated to assess variables associated to persistent use of drugs, where the outcome was defined as 1 in the case of use of drugs at two adjacent time points and 0 otherwise. In addition, exploratory analyses assessing association between change in NPI sub-syndromes and persistent use of drugs were performed. All multivariate models were reduced using Akaike’s Information Criteria, where a lower value means a better model. The results were tabulated as odds ratios (ORs) with corresponding 95% confidence intervals (CI) and presented graphically.

All analyses were performed in SPSS version 22 and SAS v9.3. *P*-values below 0.05 were considered statically significant. All tests were two-sided.

## Results

### Sample characteristics

**Fig. 1:**
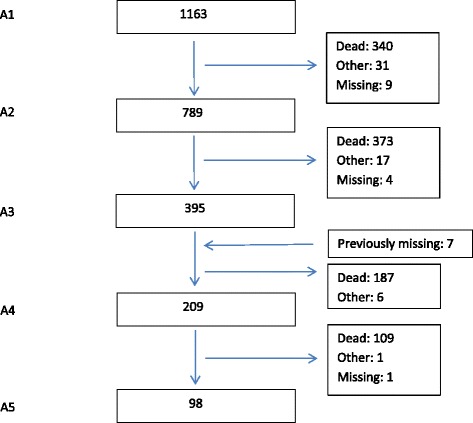
Flow chart. Mean (SD) time between accessions were 11.1 (0.5) months for A1–A2, 18.6 (1.3) months for A2–A3, 20.5 (1.7) months for A3–A4 and 20.8 (2.8) months for A4–A5

At baseline, the mean (SD) age of the residents was 84.4 (7.8) years and 846 (72.7%) of them were women (Table 1). The mean (SD) baseline CDR sum of boxes was 11.2 (5.3) and 932 (80.1%) residents had CDR ≥ 1 indicating dementia. The mean length of stay at baseline was 938.3 (1013.1) days and the mean (SD) number of prescribed drugs taken regularly was 6.0 (3.1). Of the 1163 residents at baseline, 98 (8.4%) were still alive and available at the fifth follow-up (see Fig. 1). Mean (SD) time of follow-up was 829.5 (690.0) days.

**Table 1:** Sample characteristics at baseline

		A1	CDR ≥ 1	CDR < 1	*P*-values^a^
		1163	932		
Socio-demographics					
Age	Mean (SD)	84.4 (7.8)	84.5 (7.5)	83.8 (9.0)	0.223
Females	N (%)	846 (72.7)	686 (73.6)	156 (68.7)	0.139
Education < 10 years	N (%)	847 (74.8)	673 (74.1)	170 (76.9)	0.390
Married	N (%)	221 (19.0)	185 (19.8)	36 (15.9)	0.178
Health condition					
GMHR					
Good Fairly good Poor Very Poor	N (%) N (%) N (%) N (%)	194 (17.2) 386 (34.1) 378 (33.4) 173 (15.3)	142 (15.7) 291 (32.2) 322 (35.6) 150 (16.6)	52 (23.4) 94 (42.3) 53 (23.9) 23 (10.4)	<0.001
PSMS score	Mean (SD)	18.1 (5.4)	18.8 (5.3)	15.4 (4.8)	<0.001
NPI Agitation sub-syndrome	Mean (SD)	5.8 (8.0)	6.5 (8.2)	2.9 (6.1)	<0.001
NPI Psychosis sub-syndrome	Mean (SD)	2.8 (5.1)	3.2 (5.3)	1.3 (3.8)	<0.001
NPI Affective sub-syndrome	Mean (SD)	3.5 (5.3)	3.7 (5.4)	2.9 (4.9)	0.041
NPI Apathy	Mean (SD)	2.2 (3.7)	2.4 (3.8)	1.1 (2.9)	<0.001
No of drugs	Mean (SD)	6.0 (3.1)	5.8 (3.0)	6.9 (3.3)	<0.001
Days in NH	Mean (SD)	938.3 (1013.0)	928.9 (910.1)	975.5 (1359.3)	0.534

*CDR* Clinical Dementia Rating scale

*GMHR* General Medical Health rating

*PSMS* Physical Self-Maintenance Scale

*NPI* Neuropsychiatric Inventory

*NH* Nursing home

^a^Calculated by using t-test for Independent samples for continuous or χ^2^-test for categorical variables

### Prevalence of psychotropic drugs over time

The prevalence of PTD use at baseline and each of the follow-up time points are presented in Table 2. Antidepressants were most frequently used (38.3% at baseline, 32.7% at last follow-up); while anti-dementia drugs were least frequently used (11.3% at baseline, 0% at last follow-up). The prevalence of use of any PTD was high throughout the period; at baseline the prevalence was 72.9%, but had fallen to 63.3% at the last follow-up.

**Table 2:** Prevalence and persistence of PTD use according to the presence of dementia at each assessment (%)

	Prevalence in percentages									
	A_1_		A_2_		A_3_		A_4_		A_5_	
	All (*n* = 1163)	D/nD (*n* = 932/227)	All (*n* = 789)	D/nD (*n* = 628/159)	All (*n* = 395)	D/nD (*n* = 300/94)	All (*n* = 209)	D/nD (*n* = 160/48)	All (*n* = 98)	D/nD (*n* = 75/22)
Antipsychotics (AP)	24.1	26.0/15.9***	25.2	26.4/20.8	20.5	22.0/16.0	20.6	22.5/14.6	20.4	20.0/22.7
Trad. AP	11.3	11.5/11.0	12.2	11.6/14.5	9.4	9.0/10.6	8.6	9.4/6.3	12.2	12.0/13.6
Atypical AP	13.2	14.9/5.7***	13.8	15.6/6.9***	11.9	13.7/6.4**	12.6	13.1/8.3	8.2	8.0/9.1
Antidepressants	38.3	39.1/34.8	37.3	37.9/34.0	36.7	35.3/40.4	35.4	33.1/41.7	32.7	29.3/40.9
Anxiolytics	24.2	23.8/26.4	24.2	22.9/29.6*	25.1	21.3/37.2***	28.7	26.9/35.4	22.4	21.3/27.3
Sedatives	29.0	26.4/39.6***	26.6	23.4/38.4***	24.1	21.0/33.0*	23.9	19.4/37.5**	23.5	20.0/31.8
Antidementia drug	11.3	13.5/2.2***	9.8	11.8/1.9***	5.3	6.3/2.1*	2.9	3.1/2.1	0	0/0
Any PTD	72.9	74.5/66.5*	71.4	71.5/70.4	70.4	67.7/78.7*	69.4	66.9/77.1	63.3	57.3/81.8**
	Persistence at two consecutive time points in percentages									
			A_1_–A_2_		A_2_–A_3_		A_3_–A_4_		A_4_–A_5_	
			All	D/nD	All	D/nD	All	D/nD	All	D/nD
Antipsychotics (AP)			76.7	75.2/88.9*	64.9	67.6/55.0	69.6	67.5/83.3	70.0	68.8/75.0
Trad. AP			73.6	70.8/84.2	61.4	63.3/57.1	73.7	73.3/75.0	80.0	75.0/100
Atypical AP			74.5	74.7/80.0	61.5	63.0/50.0	62.1	59.3/100***	60.0	62.5/50.0
Antidepressants			80.4	79.8/82.8	76.5	73.1/87.9*	72.2	66.1/89.5**	75.7	76.9/70.0
Anxiolytics			76.9	73.6/89.5**	69.2	62.7/84.4**	86.8	82.1/100**	66.7	66.7/66.7
Sedatives			70.9	65.8/82.8**	64.8	69.1/55.6	69.6	64.9/77.8	68.0	58.8/85.7
Antidementia drug			66.0	65.3/100***	38.1	36.6/100***	27.3	27.3/0	0	0/0
Any PTD			89.8	88.7/94.3*	85.3	82.4/94.2**	89.3	86.6/97.3**	78.4	75.5/85.0

A1–A5: Assessment 1–5

*D* Dementia CDR ≥ 1; *nD* No dementia CDR < 1

CDR ratings were missing for 4 people at A_1_, 2 people at A_2_ and 2 people at A_3_

*CDR* Clinical Dementia Rating scale

*Trad* Conventional

*PTD* Psychotropic drugs

*p < 0.05, **p ≤ 0.01; ***p ≤ 0.001 (Z-test for proportions used)

At baseline, the use of any PTD occurred more frequently in residents with dementia, while at the last follow-up any PTD was more often used in residents without dementia. Atypical antipsychotics were used more frequently in residents with dementia at A1–A3, whereas residents without dementia used anxiolytics more frequently at A2 and A3 and sedatives more frequently at A1–A4, respectively.

### Persistent use of psychotropic drugs

**Fig. 2:**
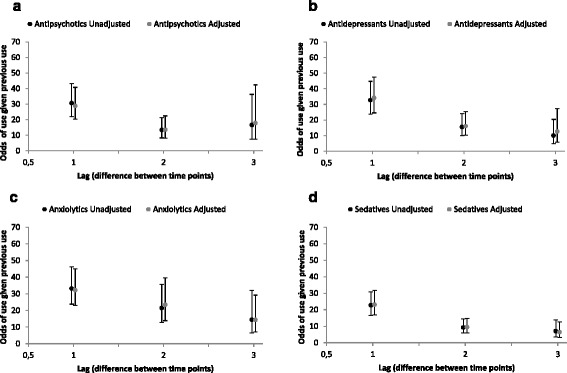
Illustration of OR for use of each category of psychotropic drugs at one time point given use of the same category of psychotropic drug at an earlier time point by distance (Lag) between the time points, unadjusted and adjusted. **a** Antipsychotics; **b** Antidepressants; **c** Anxiolytics; and **d** Sedatives

The proportion of PTD use at two consecutive time points was high (>50%) throughout the period for all types of PTD, except for use of anti-dementia drugs (Table 2). Persistent use of anxiolytics at two consecutive time points was higher for residents without dementia than those with dementia. Among those who completed all assessments (*n* = 98), 10.4% used antipsychotic drugs, 19.8% used antidepressants, 11.5% used anxiolytics and 9.4% used sedatives at all assessments. Unadjusted and adjusted odds for use of PTD at one time point, given use of the same type of PTD at an earlier time point was estimated for all types of PTD except for anti-dementia drug use (Table 3 and Fig. 2). Both in unadjusted and adjusted analyses, the odds for persistent use of antipsychotics, antidepressants, anxiolytics and sedative were high. The odds for persistent use of these PTD were highest when compared with use at the closest earlier assessment time point (Lag 1) and fell successively when the distance between the assessment time points increased (Lags 2 and 3), with one exception for antipsychotics where the odds slightly increased when there were three time points between assessments (Lag 3) compared to two time points (Lag 2). All results were highly significant (*p* < 0.001).

**Table 3:** Odds ratios for use of each category of psychotropic drugs at one time point given use of the same category of the psychotropic drug at an earlier time point (the distance between time points is called lag), unadjusted and adjusted analyses where relevant covariates adjusted for were measured at the same earlier time

Variables	Unadjusted		Adjusted	
	OR (95% CI)	*P*-value	OR (95% CI)	*P*-value
Lag 1, *N* = 1406 observations				
Antipsychotics^a^	30.7 (21.8; 43.1)	<0.001	28.8 (20.31; 40.8)	<0.001
Antidepressants^b^	32.6 (23.8; 44.8)	<0.001	34.1 (24.6; 47.4)	<0.001
Anxiolytics^c^	33.1 (23.7; 46.2)	<0.001	32.2 (23.1; 45.0)	<0.001
Sedatives^d^	22.6 (15.6; 30.8)	<0.001	23.1 (16.8; 31.7)	<0.001
Lag 2, *N* = 654 observations				
Antipsychotics^e^	13.3 (8.3; 21.4)	<0.001	13.5 (8.1; 22.3)	<0.001
Antidepressants^f^	15.5 (9.9; 24.2)	<0.001	16.0 (10.2; 25.3)	<0.001
Anxiolytics^g^	21.4 (12.8; 35.7)	<0.001	23.4 (13.9; 39.6)	<0.001
Sedatives^h^	9.3 (5.9; 14.4)	<0.001	9.4 (5.9; 14.8)	<0.001
Lag 3, *N* = 288 observations				
Antipsychotics^i^	16.5 (7.5; 36.3)	<0.001	17.9 (7.5; 42.4)	<0.001
Antidepressants^j^	10.0 (4.9; 20.4)	<0.001	12.6 (5.8; 27.2)	<0.001
Anxiolytics^k^	14.4 (6.5; 32.1)	<0.001	14.3 (7.0; 29.2)	<0.001
Sedatives^l^	7.0 (3.6; 13.9)	<0.001	6.4 (3.2; 12.6)	<0.001

Lag 1: two consecutive assessment time points

Lag 2: one time point between selected time points

Lag 3: two time points between selected time points

^a^Adjusted for: PSMS score, NPI Agitation sub-syndrome, NPI Psychosis sub-syndrome, NPI Affective sub-syndrome, NH size age and duration in NH

^b^Adjusted for: PSMS score, NPI- Affective sub-syndrome, NPI Apathy and duration in NH

^c^Adjusted for CDR sum of boxes, NPI- Affective sub-syndrome and level of education

^d^Adjusted for PSMS score, NPI Apathy and duration in NH

^e^Adjusted for PSMS score, NPI Psychosis sub-syndrome, NPI Apathy, Type of nursing home and age

^f^Adjusted for: CDR sum of boxes, PSMS score and NPI- Affective sub-syndrome

^g^Adjusted for CDR sum of boxes, PSMS score and level of education

^h^Adjusted for CDR sum of boxes, PSMS and age

^i^Adjusted for PSMS score and age

^j^Adjusted for: CDR sum of boxes, NPI- Affective sub-syndrome and NPI Apathy

^k^Adjusted for NPI Agitation sub-syndrome, NPI Psychosis sub-syndrome, NPI Affective sub-syndrome, NPI Apathy, level of education and Type of nursing home unit

^l^Adjusted for CDR sum of boxes, NPI Psychosis sub-syndrome and Nursing home size

### Factors associated with use of psychotropic drugs at two consecutive time points

The adjusted risk for persistent use of antipsychotics at two consecutive time points was elevated when residents had higher psychosis sub-syndrome score or were younger, were male or had a longer NH stay at baseline (Table 4). The adjusted risk for persistent use of antidepressants was elevated when residents had higher P-ADL functioning (lower PSMS score) or higher affective sub-syndrome score. The adjusted risk for persistent use of anxiolytics was elevated when residents had higher P-ADL functioning (lower PSMS score) or higher affective sub-syndrome score or stayed in a larger NH (Table 5). The adjusted risk for persistent use of sedatives was elevated when residents had better cognitive functioning (lower CDR sum of boxes) or higher affective sub-syndrome score or lower apathy symptom score.

**Table 4:** Odds of use of Antipsychotics or Antidepressants at one time point given use of Antipsychotics or Antidepressants at the previous time point, covariates measured at the “previous” time point, *N* = 1406 observations

Variables	Antipsychotics				Antidepressants			
	Unadjusted		Adjusted		Unadjusted		Adjusted	
	OR (95% CI)	*P*-value	OR (95% CI)	*P*-value	OR (95% CI)	*P*-value	OR (95% CI)	*P*-value
Assessed at previous time point								
CDR sum of boxes	1.04 (1.002; 1.07)	0.036			0.97 (0.95; 0.995)	0.020		
GMHR								
Good	1.25 (0.66; 2.37)	0.498			0.98 (0.59; 1.63)	0.934	0.63 (0.35; 1.12)^3^	0.111
Fairly good	1.11 (0.63; 1.98)	0.715			1.29 (0.82; 2.02)	0.273	0.92 (0.56; 1.51)^8^	0.745
Poor	1.53 (0.86; 2.72)	0.151			1.62 (1.03; 2.56)	0.038	1.34 (0.83; 2.16)^5^	0.229
Very Poor	1	-			1	-	1	-
PSMS score	1.03 (0.997; 1.06)	0.073			0.96 (0.94; 0.98)	0.001	0.94 (0.91; 0.97)^2^	<0.001
NPI Agitation sub-syndrome	1.05 (1.03; 1.07)	<0.001	1.02 (0.997; 1.04)^6^	0.093	0.997 (0.98; 1.01)	0.714		
NPI Psychosis sub-syndrome	1.09 (1.06; 1.12)	<0.001	1.06 (1.03; 1.10)^2^	0.001	1.00 (0.98; 1.03)	0.915	0.97 (0.95; 1.00)^4^	0.060
NPI Affective sub-syndrome	1.06 (1.03; 1.09)	<0.001	1.03 (0.999; 1.07)^5^	0.060	1.08 (1.05; 1.10)	<0.001	1.08 (1.05; 1.11)^1^	<0.001
NPI Apathy	1.07 (1.03; 1.12)	<0.001			0.99 (0.96; 1.03)	0.732		
Assessed at baseline								
Age	0.95 (0.93; 0.97)	<0.001	0.95 (0.93; 0.97)^1^	<0.001	0.99 (0.98; 1.01)	0.317	0.98 (0.97; 1.00)^6^	0.056
Females	0.56 (0.40; 0.80)	0.001	0.64 (0.44; 0.93)^4^	0.019	1.38 (1.02; 1.87)	0.035	1.33 (0.97; 1.82)^7^	0.078
Education (<=10 years)	0.97 (0.68; 1.39)	0.865			0.88 (0.66; 1.17)	0.385		
Duration in NH (LN)	1.21 (1.06; 1.40)	0.007	1.21 (1.05; 1.40)^3^	0.011	0.98 (0.88; 1.09)	0.654		
Nursing home size	0.998 (0.993; 1.003)	0.382			1.00 (0.998; 1.01)	0.347		
Type of nursing home unit								
RU	1	-			1	-		
REU	0.57 (0.10; 3.41)	0.539			1.03 (0.30; 3.51)	0.959		
SCU	1.24 (0.78; 1.97)	0.367			0.80 (0.55; 1.17)	0.248		
OU	3.25 (0.63; 16.81)	0.160			1.13 (0.28; 4.61)1	0.865		

*RU* Regular units

*REU* rehabilitation unit

*SCU* special care unit for people with dementia

*OU* other units

The relative importance of each covariate in the adjusted models is included with a number after the 95% CI; lowest number has highest importance

**Table 5:** Odds of use of Anxiolytics or Sedatives or at one time point given use of Anxiolytics or Sedatives at a previous time point, covariates measured at the “previous” time point, *N* = 1406 observations

Variables	Anxiolytics				Sedatives			
	Unadjusted		Adjusted		Unadjusted		Adjusted	
	OR (95% CI)	*P*-value	OR (95% CI)	*P*-value	OR (95% CI)	*P*-value	OR (95% CI)	*P*-value
Assessed at previous time point								
CDR sum of boxes	0.96 (0.94; 0.99)	0.015	0.97 (0.94; 1.01)^4^	0.125	0.94 (0.91; 0.97)	<0.001	0.94 (0.91; 0.97)^1^	<0.001
GMHR								
Good	1.31 (0.68; 2.54)	0.421			1.20 (0.65; 2.23)			
Fairly good	1.56 (0.87; 2.81)	0.138			1.42 (0.82; 2.47)	0.560		
Poor	1.83 (1.01; 3.30)	0.046			1.43 (0.82; 2.49)	0.209		
Very Poor	1	-			1	0.212		
PSMS score	0.96 (0.93; 0.98)	0.002	0.96 (0.93; 0.99)^3^	0.016	0.95 (0.93; 0.98)	-		
NPI Agitation sub-syndrome	1.00 (0.98; 1.02)	0.964			0.99 (0.97; 1.01)	0.001		
NPI Psychosis sub-syndrome	1.01 (0.98; 1.04)	0.451			1.00 (0.97; 1.04)	0.358		
NPI Affective sub-syndrome	1.08 (1.05; 1.11)	<0.001	1.09 (1.06; 1.12)^1^	<0.001	1.04 (1.01; 1.07)	0.878 0.005	1.06 (1.03; 1.09)^2^	<0.001
NPI Apathy	1.01 (0.97; 1.05)	0.662			0.94 (0.90; 0.99)	0.012	0.95 (0.90; 0.996)^4^	0.034
Assessed at baseline								
Age	0.997 (0.98; 1.02)	0.775			1.002 (0.98; 1.02)	0.812		
Females	1.01 (0.71; 1.44)	0.967			1.28 (0.89; 1.83)	0.182		
Education (<=10 years)	1.16 (0.81; 1.66)	0.416			0.82 (0.59; 1.15)	0.253	0.77 (0.54; 1.08)^5^	0.125
Duration in NH (LN)	1.07 (0.94; 1.22)	0.292	1.12 (0.98; 1.28)^5^	0.101	0.91 (0.81; 1.04)	0.164		
Nursing home size	1.01 (1.002; 1.01)	0.004	1.007 (1.002; 1.011)^2^	0.002	1.00 (0.999; 1.01)	0.109	1.004 (1.00; 1.008)^3^	0.072
Type of nursing home unit								
RU	1	-			1	-		
REU	0.18 (0.02; 1.83)	0.146			2.35 (0.63; 8.79)	0.206		
		0.754			0.62 (0.39; 0.98)	0.041		
SCU	0.93 (0.58; 1.49)							
OU	2.96 (0.54; 16.13)	0.209			1.67 (0.34; 8.19)	0.526		

*RU* Regular units

*REU* rehabilitation unit

*SCU* special care unit for people with dementia

*OU* other units

The relative importance of each covariate in the adjusted models is included with a number after the 95% CI; lowest number has highest importance

In a subsequent analysis where sub-syndrome scores of NPS at the first time point were replaced by change in sub-syndrome score between the two assessments, we found no association between change in the sub-syndrome scores of NPS and persistent use of antipsychotic drugs, antidepressants, anxiolytics or sedatives.

## Discussion

In this Norwegian NH study, the prevalence and persistent use of PTD at two consecutive time points was high, both for residents with and without dementia, except for use of an anti-dementia drug. Close to three-quarters of the dementia and two-thirds of non-dementia residents used PTD at baseline of the data collection. Persistent use of anxiolytics was more common in residents without dementia. The persistent use decreased gradually when the distance between the assessment time points increased (Lags 2 and 3), with the exception of the use of antipsychotics drugs. More severe NPS were associated with persistent use of antipsychotics drugs, antidepressants, anxiolytics and sedatives at the next time point, but change in NPS between the time points was not associated with persistent use of PTD. Better P-ADL functioning (lower PSMS score) was associated with persistent use of antidepressants and anxiolytics. Furthermore, less severe dementia was associated with persistent use of sedatives. Of the organizational variables included in the analysis, we found that a longer stay in NH increased the odds for persistent use of antipsychotics at two consecutive time points and staying in a larger-sized NH increased the odds for persistent use of anxiolytics.

### Use of antipsychotics

The study found that the prevalence of atypical antipsychotics was higher in residents with dementia than in residents without, but only for the first three time points. Use of conventional antipsychotics did not differ between residents with or without dementia at any time point. The persistence in use of both atypical and conventional antipsychotics at two consecutive time points was high (>50%) during the entire follow-up period of 72 months for both groups of residents. A small 6-month follow-up study of newly arrived NH residents in Australia has previously reported the persistence of antipsychotics to be equally high [49]. Given the strong evidence on the increased mortality risk associated with use of conventional antipsychotics in people with dementia it is rather surprising to see that they are still used to that extent. These findings are alarming, since the duration of such treatment should be as short as possible [32]. In the present adjusted logistic regression analysis for persistent use of antipsychotic drugs at two consecutive time points, higher severity of psychosis increased the risk for persistent use of antipsychotics. However, it is surprising that in the exploratory analysis, a change in NPS was not associated with persistent use of antipsychotics. Clinical recommendations have highlighted the need for clinicians to monitor NPS closely and consider discontinuing treatment with antipsychotics when an obvious treatment effect does not occur or the residents have side effects due to the treatment [32]. It could be that people receiving PTD had more severe symptoms prior to drug initiation. Also, it could be that antipsychotics were described as unspecific sedatives. Very few studies have examined variables associated with persistent use of antipsychotics in NH residents [46, 52], but none of these studies have explicitly explored the importance of NPS in the persistent use of antipsychotic drugs. However, cross-sectional studies have explored the association between NPS and use of antipsychotic drugs and found that higher total symptom load [80] and more severe psychosis sub-syndromes symptoms were associated with use of antipsychotics [11, 14, 81, 82].

In the adjusted logistic regression analysis of use of antipsychotics, younger age and male gender increased the risk for persistent use of antipsychotics. In contrast, a small Swedish 6-month follow-up study in NH residents with dementia did not find age, gender or other personal characteristics of the residents such as P-ADL or cognitive functioning important for persistent use of antipsychotics [52]. Our finding may partly be explained by age and gender-based expressions of behavioral symptoms [80] not captured by the NPS. Male and younger residents may be experienced as more threatening in their verbal or physical expressions and are physically stronger compared to women and older residents, and for this reason may be put on antipsychotic drugs. In line with our results, cross-sectional studies of use of antipsychotics in NH residents have found that younger residents [4, 8, 15, 22, 80, 82, 83] and male residents [5, 80, 84] are more likely to receive antipsychotics.

Persistent use of antipsychotics has been reported to be more frequent in regular care units than in SCU [46]. In the present study we did not find an association between the type of care unit and persistent use of antipsychotics. In Norway, SCUs have residents with more severe NPS than other units, which could explain our result [30]. However, we found that residents with a longer stay in NH at baseline had an increased likelihood of being persistent users of antipsychotics. We do not have a firm explanation for this, but it may be that newly arrived NH residents receive more attention from the staff or respond better to care that is intended to reduce stress, strain and symptoms related to dementia.

### Use of antidepressants

We found, in line with other studies, that antidepressants were the most frequently used PTD in NH residents [48, 49] and that the frequency did not differ between residents with or without dementia [85]. The persistence of use of antidepressants was also high [49]. As we could expect, those with more severe affective symptoms had higher odds for persistent use of antidepressants. This result is in line with cross-sectional studies of associations for use of antidepressants [11, 15, 81]. Even so, the efficacy of antidepressants in treating depression in persons with dementia is uncertain. The high prevalence and persistence of antidepressants use may indicate that these drugs also are used for the treatment of agitation. Better performance in P-ADL was more likely to be associated with persistent antidepressant users. We do not have a firm explanation for this, but it may be that those with better P-ADL also have a better ability to express their emotional state.

### Use of anxiolytics, sedatives and anti-dementia drugs

The prevalence of anxiolytics in the NH residents varied between 20 and 40%, and was significantly higher in residents without dementia, while the persistent use of anxiolytics in adjusted analysis was not explained by the severity of dementia. As for antidepressants, persistent use of anxiolytics was explained by more severe affective symptoms and better P-ADL functioning, but not by change in affective sub-syndrome symptoms between time points. The size of the NH was associated with persistent use of anxiolytics. We speculate that staff distress [81], registered nursing hours per resident [86] and other organizational factors [5] that we have not measured may be related to NH size, quality of care and use or persistent use of anxiolytics.

Use of sedatives was higher among residents without dementia than residents with dementia and varied between 19 and 26% in residents with dementia and between 32 and 40% in residents without dementia. A possible explanation may be that residents without dementia are more vulnerable to disturbances in the NH environment and at the same time may be more able to ask for sedatives than residents with dementia.

The prevalence of anti-dementia drugs use was at baseline 11.3% for all residents and as expected the prevalence of such use declined at each of the follow-ups and at the fifth assessment no one used anti-dementia drugs. At the four first assessment time points between 1.9 to 2.2% of residents with CDR less than 1 were prescribed anti-dementia drugs. This may indicate that the NH physicians have prescribed the anti-dementia drugs without having a dementia diagnosis (P70), but the physicians may have used the diagnosis cognitive problems (P20) as an indication for prescribing the drug. This is quite common in Norway. However, in a recent Norwegian study it was found that a substantial number of persons who purchased anti-dementia drugs had no diagnosis of dementia or cognitive problems registered in the primary health care service system [87].

### Strength and limitations

The study has significant strengths. Firstly, all nurses participating in the data collection participated in a 2-day educational course to ensure that they had adequate knowledge prior to collecting data for this study and participated in a 1-day educational course before the follow-up data collections. This ensured high data quality. Secondly, a large sample size allowed us to adjust for many potentially important variables, such as health and demographic factors. Furthermore, this study benefits from the inclusion of NHs located in a large part of the country. However, we cannot guarantee that the sample is representative for Norwegian NH residents since inclusion was not based on random selection from all NHs in Norway.

The study has some limitations. Firstly, the data from the present study is quite old since data collection started in 2004, and thus, may not represent medication use patterns in Norwegian NHs of today. Secondly, a high drop-out rate mainly due to death might have introduced some bias into the results. However, this methodological problem is inherent to most longitudinal NH studies. Also, we used linear mixed models that include all available data (data from drop-outs as well) in the analysis. Due to the low number of participants at the end of the follow-up period, the analysis of persistence was limited to three lags only. Thirdly, the time intervals between the assessments varied somewhat among participants. However, this affects the results only minimally, since the time intervals between assessments were quite long and not used in the models explicitly, only as lags. In the present study the time intervals between the assessments were 1 year or more and due to the study design, we do not know whether there were changes in PTD use between assessments.

Fourthly, there is a limitation on the accuracy of dementia diagnoses, since dementia and degree of dementia are based on the CDR rating of several assessors and the fact that a CDR assessment was used, and not a standardized dementia diagnosis, including neuropsychological tests. A large number of residents with a CDR score of 3 could not be tested with any dementia tests such as the Mini Mental Status Examination or the Clock Drawing Test. However, CDR is an accepted assessment tool and is commonly used in epidemiological NH studies to identify dementia and measure the severity of dementia [65, 88], and the agreement between CDR and a diagnostic assessment according to the ICD-10 is high [62].

## Conclusion

Psychotropic drugs were frequently used as a long-term treatment among NH residents and were associated with severity of neuropsychiatric symptoms, but not with severity of dementia. The high prevalence and persistence of psychotropic drug use may indicate that the treatment is not in line with current treatment recommendation. It is important that clinicians monitor effects and side effects of PTD treatment closely and stop treatment when the risk is not balanced by considerable benefits to the NH resident.

## Abbreviations

CDR: Clinical Dementia Rating
CI: Confidence intervals
GMHR: General Medical Health Rating
NH: Nursing home
NPI: Neuropsychiatric Inventory
NPS: Neuropsychiatric symptoms
OR: Odds ratio
OU: Other units
P-ADL: Personal Activities of Daily Living
PSMS: Physical Self-Maintenance Scale
PTD: Psychotropic drugs
REU: Rehabilitation unit
RU: Regular units
SCU: Special care unit for people with dementia
SD: Standard deviation
